# Supplementary material for: DNA methylation dynamics and dysregulation delineated by high-throughput profiling in the mouse
Source: Cell Genom. Author manuscript; Available in PMC 2022 Jul 22. (PMC9306256; doi:10.1016/j.xgen.2022.100144)
Supplement: Supplementary file 1 [file NIHMS1823589-supplement-1.pdf]

Cell Genomics, Volume *2*

Supplemental information

DNA methylation dynamics and dysregulation delineated by high-throughput profiling in the mouse

Wanding Zhou, Toshinori Hinoue, Bret Barnes, Owen Mitchell, Waleed Iqbal, Sol Moe Lee, Kelly K. Foy, Kwang-Ho Lee, Ethan J. Moyer, Alexandra VanderArk, Julie M. Koeman, Wubin Ding, Manpreet Kalkat, Nathan J. Spix, Bryn Eagleson, John Andrew Pospisilik, Piroska E. Szabó, Marisa S. Bartolomei, Nicole A. Vander Schaaf, Liang Kang, Ashley K. Wiseman, Peter A. Jones, Connie M. Krawczyk, Marie Adams, Rishi Porecha, Brian H. Chen, Hui Shen, and Peter W. Laird

Supplemental Figures

RESOURCE:

DNA Methylation Dynamics and Dysregulation Delineated by High-Throughput Profiling in the Mouse

Wanding Zhou[1,2]*, Toshinori Hinoue[3], Bret Barnes[4], Owen Mitchell[3], Waleed Iqbal[1], Sol Moe Lee[1], Kelly K. Foy[3], Kwang-Ho Lee[3], Ethan J. Moyer[1], Alexandra VanderArk[5], Julie M. Koeman[6], Wubin Ding[1], Manpreet Kalkat[3], Nathan J. Spix[3], Bryn Eagleson[7], John Andrew Pospisilik[3], Piroska E. Szabó[3], Marisa S. Bartolomei[8], Nicole A. Vander Schaaf[3,§], Liang Kang[3], Ashley K. Wiseman[3], Peter A. Jones[3], Connie M. Krawczyk[5], Marie Adams[6], Rishi Porecha[4], Brian H. Chen[9], Hui Shen[3]*, Peter W. Laird[3,10]*

[1]Center for Computational and Genomic Medicine, Children's Hospital of Philadelphia, PA, 19104, USA
[2]Department of Pathology and Laboratory Medicine, University of Pennsylvania, Philadelphia, PA, 19104, USA
[3]Department of Epigenetics, Van Andel Institute, Grand Rapids, MI 49503, USA
[4]Illumina, Inc., Bioinformatics and Instrument Software Department, San Diego, CA 92122, USA
[5]Department of Metabolism and Nutritional Programming, Van Andel Institute, Grand Rapids, MI 49503, USA
[6]Genomics Core, Van Andel Institute, Grand Rapids, MI 49503, USA
[7]Vivarium and Transgenics Core, Van Andel Institute, Grand Rapids, MI 49503, USA
[8]Department of Cell and Developmental Biology, Epigenetics Institute, University of Pennsylvania Perelman School of Medicine, Philadelphia, PA 19104, USA
[9]FOXO Technologies Inc., Minneapolis, MN 55402, USA

*Correspondence:
wanding.zhou@pennmedicine.upenn.edu, hui.shen@vai.org, peter.laird@vai.org

[10]Lead Contact: Peter W. Laird, peter.laird@vai.org

[§]Current Address: Department of Biological Sciences, Olivet Nazarene University, Bourbonnais, IL 60914, USA

Supplemental Figure S1 - Related to Figure 1

A

DESIGN GROUP	DESIGN INTENT OR STRATEGY	ABBREVIATION	N	%
**Genomic Features**				
Promoters	Gene promoter CpGs within 1500bp flanking TSSs (Coding genes)	TSS	100948	34.1%
	Gene promoter CpGs within 1500bp flanking TSSs (LncRNAs)	lincRNATSS	15030	5.1%
	Gene promoter CpGs within 1500bp flanking TSSs (Pseudogenes)	PseudogeneTSS	10339	3.5%
	CpGs within 500bp flanking miRNAs	miRNA	4222	1.4%
Enhancers	CpGs in enhancer elements from diverse tissues (mouse ENCODE)	Enhancer	58759	19.8%
	CpGs in enhancer elements (VISTA database)	EnhancerVista	1247	0.4%
	CpGs located at or close to CTCF binding sites	CTCF	8616	2.9%
Non-Promoter CpG Islands	CpGs in non-promoter CpG islands	CGI	17134	5.8%
Gene Body CpGs	Gene body CpGs at least 2000bp downstream from TSSs	GeneBody	111702	37.7%
Heterochromatin	Probes targeting CpGs in multi-copy transposable elements	RMSK	4723	1.6%
Repeat CpGs	Designed multimapping probes	Multi	7317	2.5%
Chromosome X, Y	CpGs located in the mouse X chromosome	X	15174	5.1%
	CpGs located in the mouse Y chromosome	Y	3780	1.3%
Mitochondria	CpGs located in the mouse mitochondrial genome	Mitochondria	32	0.0%
**Target Biology**				
Imprinting & Mono-Allelic Methylation	CpGs with putative mono-allelic methylation (adult tissues)	MonoallelicMeth	7813	2.6%
	CpGs in imprinting-associated differentially methylated regions	ImprintDMR	654	0.2%
	CpGs with putative mono-allelic methylation (placenta)	PlacentaIntermed	981	0.3%
Germ Cell Development	CpGs specifically unmethylated in oocytes	OocyteUnmeth	486	0.2%
	CpGs specifically methylated in oocytes	OocyteMeth	172	0.1%
	CpGs specifically methylated in primordial germ cells	PGCMeth	474	0.2%
	CpGs specifically methylated in sperm	SpermMeth	397	0.1%
	CpGs specifically unmethylated in sperm	SpermUnmeth	208	0.1%
Early Embryonic Development	CpGs specifically methylated mouse placenta	PlacentaMeth	485	0.2%
	CpGs specifically unmethylated in mouse placenta	PlacentaUnmeth	484	0.2%
	CpGs specifically methylated in zygotes	ZygoteMeth	474	0.2%
	CpGs specifically unmethylated in zygotes	ZygoteUnmeth	480	0.2%
Epigenetic Clock, Aging and Cancer	CpGs hypermethylated in intestinal adenomas	Adenoma	8330	2.8%
	CpGs whose methylation was found predictive of epigenetic age	Clock	765	0.3%
	CpGs in common PMD and in solo-WCGW context	PMDsoloWCGW	5095	1.7%
Metastable Epi-alleles	CpGs at candidate metastable epialleles	VMR	5849	2.0%
Human-mouse Synteny	CpGs in synteny with human genome CpGs included in the human EPIC array	EPIC	29054	9.8%
**Random Selection**				
Random CpGs	Randomly-selected CpGs (sex-chromosomes over-sampled)	Random	28011	9.5%
Mouse Strain SNPs	Strain-distinguishing SNPs (N=591) for 35 common inbred strains	SNP	1485	0.5%
**Non-CpG And Other Probe Selection**				
CpH Methylation	Probes to target non-CpG cytosine methylation	CpH	2310	0.8%
Technical Controls	Control probes to assess proper probe hybridization and extension	Control	2874	1.0%
Noninformative Probes	Probes with design flaws (unknown probes)	UK	4541	1.5%
Total Number (Overlaps Removed)		Sum	**296070**	100%

B

Array	#CpGs	%CpGs
HM450	482421	1.6%
EPIC	862927	2.9%
Mouse	284860	1.3%

C

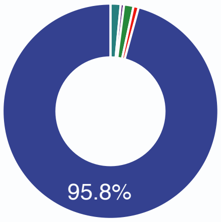

Type	HM450	EPIC	Mouse
CpG	482421	862927	284860
CpH	3091	2932	2310
SNP	65	59	1485
Control	850	635	2874
Other	0	0	4541
Total	486427	866553	296070

D

Type	Infinium	HM450		EPIC		Mouse	
CpG	I	135476	28%	142137	16%	61873	22%
	II	346945	72%	720790	84%	222987	78%
	Total	482421		862927		284860	

E

CATEGORY	N
BS	1049
Extension	2
GT	9
Hyb	4
Negative	416
NG	395
Norm	171
NP	823
Restore	1
Specificity	3
Target	1

F

		CpG		CpH		SNP	
	Strand	Converted	Synthesized	Converted	Synthesized	Converted	Synthesized
HM450	Watson	241591	0	1558	0	32	0
	Crick	240800	0	1533	0	33	0
EPIC	Watson	431992	0	1482	0	29	0
	Crick	430897	0	1450	0	30	0
Mouse	Watson	141597	887	0	522	715	18
	Crick	141465	911	1788	0	731	21

G

	HM450		EPIC		Mouse	
	%High Mapping Quality (MapQ >30)	N	%High Mapping Quality (MapQ >30)	N	%High Mapping Quality (MapQ >30)	N
CpG*	93%	482421	94%	862927	99.2%	284860
CpH	90%	3091	90%	2932	99.6%	2310
SNP	100%	65	100%	59	99.5%	1485

* Excludes 7364 probes designed to target transposable elements and repeat regions.

	Mouse	
	%High Mapping Quality (MapQ >30)	N
CpG (designed multi-mapping)	0.4%	7364
Control	36.6%	2874
Other* (designed unique)	98.6%	1543
Other* (designed multi-mapping)	2.8%	2998

* - "uk" probes

H

			mm39			
	mm10	MAPQ	[0,10]	(10,30]	(30,59]	(59,60]
CpG	type IA	[0,10]	1951	1	0	0
		(10,30]	2	544	0	0
		(30,59]	5	1	3417	0
		(59,60]	5	1	4	55944
	type IB	[0,10]	1963	1	0	0
		(10,30]	3	581	0	0
		(30,59]	5	1	3404	0
		(59,60]	4	1	4	55908
	type II	[0,10]	4607	1	0	4
		(10,30]	6	2286	0	1
		(30,59]	11	2	10062	4
		(59,60]	19	3	4	205968
CpH	type II	[0,10]	2	0	0	0
		(10,30]	0	8	0	0
		(30,59]	0	0	37	0
		(59,60]	1	0	0	2262

I

#Probe(s) / Site	CpG	CpH	SNP
1	278390	2310	36
2	1744	0	260
3	330	0	251
4	159	0	44
5	66	0	0
6	89	0	0
7	28	0	0
8	37	0	0
#Sites	280843	2310	591

Figure S1. Mouse DNA Methylation BeadArray Content, Related to Figure 1. (A) Design categories of the mouse Infinium BeadChip array. **(B)** Comparison of three Infinium methylation BeadChips in the number of targeted CpGs. **(C)** Number of probes with different targets in HM450, EPIC, and mouse arrays**. (D)** Number of Infinium-I vs Infinium-II comparing HM450, EPIC, and mouse arrays. **(E)** Control probes and their design categories in the Infinium Mouse Methylation Beadchip. (**F)** Comparison of the mouse array with HM450 and EPIC array in terms of converted vs synthesized strand probe design. **(G)** Comparison of the mouse array with HM450 and EPIC array in probe mappability. **(H)** Summary of the mouse array probes mapped to mm10 vs mm39. **(I)** Probe redundancy for the mouse methylation BeadChip probes.

Supplemental Figure S2 - Related to Figure 1

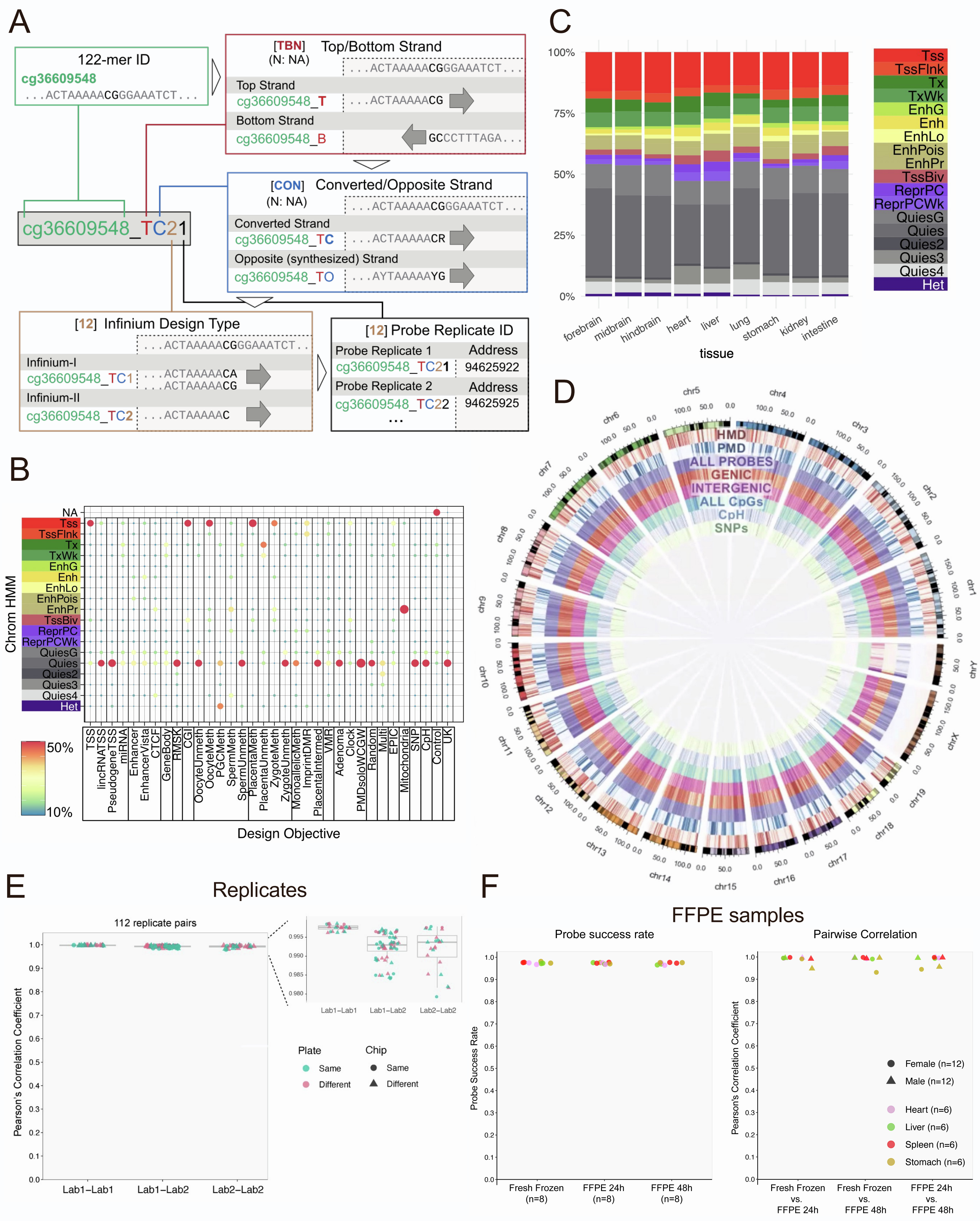

Figure S2. Mouse DNA Methylation BeadArray Probe Design and Reproducibility, Related to Figure 1. (A) Mouse array probe ID system illustration. **(B)** Enrichment of design category with chromatin state. The enrichment is consistent with the design objective with most of the TSS, CGI probes enriching for Tss chromatin state with the other probes largely falling into quiescent chromatin and heterochromatin. **(C)** Mouse array probe distribution in different chromatin states from different tissue types. **(D)** Circus plot showing distribution of mouse array-targeted CpGs in the mouse genome. **(E)** Boxplot showing pairwise Pearson's correlation coefficients within the same lab (left) and between different labs (right) **(F)** Left: Probe success rate boxplot comparing fresh frozen (FF) samples and Formalin-fixed and Paraffin-Embedded (FFPE) samples treated for 24 and 48 hours. Right: Boxplot showing pairwise correlation coefficient between FF and FFPE samples and between FFPE 24h and 48h samples.

Supplemental Figure S3 - Related to Figure 2

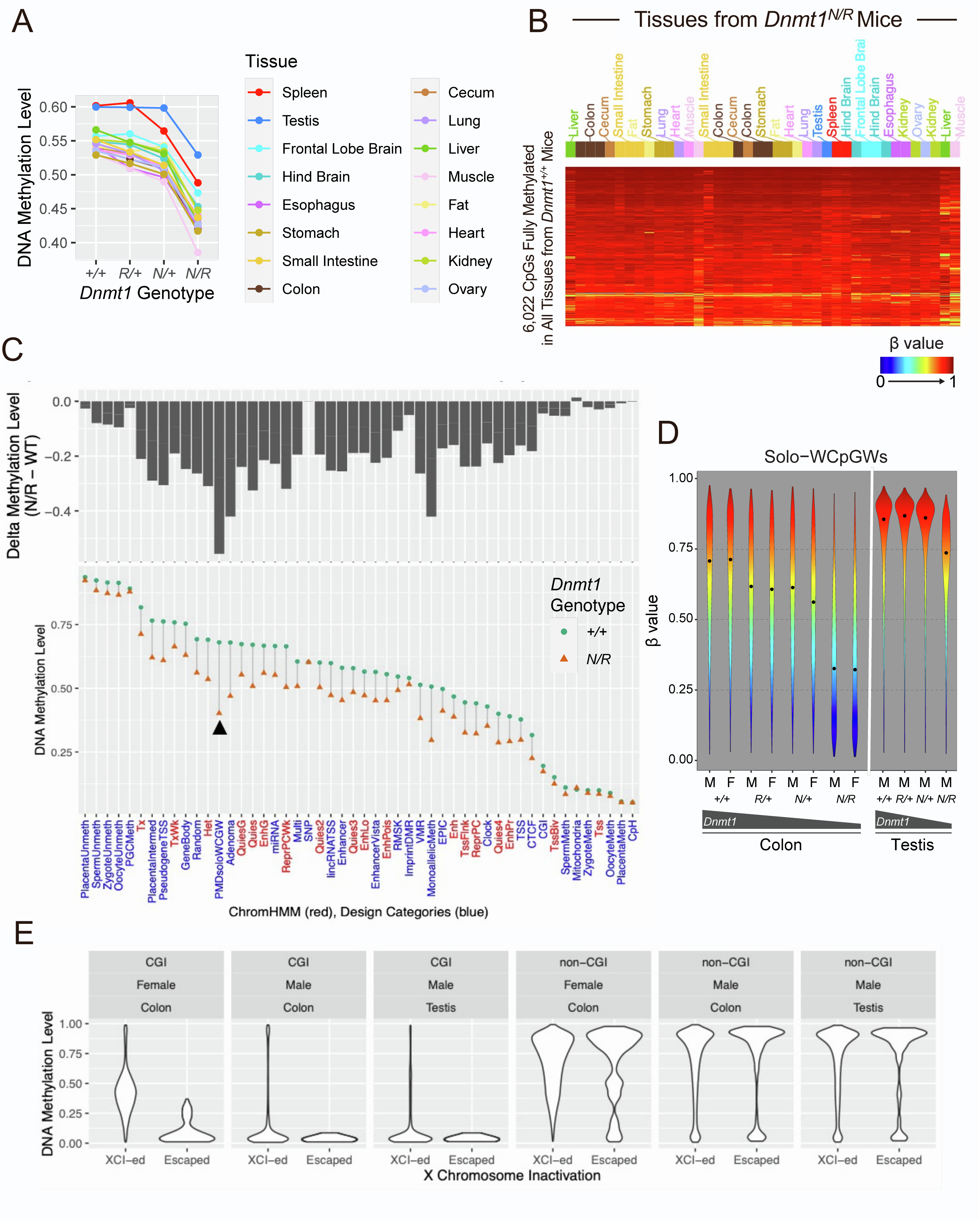

Figure S3. Experimental and Biological Validation of DNA Methylation, Related to Figure 2. **(A)** Line plot showing mean DNA methylation level across CpGs in different primary tissue samples from mice with different *Dnmt1* genotypes (X-axis). Each dot represents the median methylation level across samples of the same tissue type (color). **(B)** Retention of methylation in tissues from *Dnmt1*$^{N/R}$ mice at 6,022 CpGs that are fully methylated across all tissues in *Dnmt1*$^{+/+}$ mice. **(C)** DNA methylation level reduction in *Dnmt1*$^{N/R}$ mice compared to the wild-type mice, contrasting CpGs of different chromatin states (as characterized by chromHMM) and design categories (X-axis). The top panel shows the methylation level difference between mice of the two genotypes. The low panel shows the actual mean methylation fraction of CpGs in each category. **(D)** Distribution of Solo-WCpGW methylation in mouse colon and testis tissues comparing tissue type, sex, and four *Dnmt1* genotypes. Dots represent the mean solo-WCGW methylation level. The wedge indicates the expected trend of DNA methylation level change. **(E)** Methylation level distribution of X-linked CpGs in colon samples from male and female mice and testis samples from male mice. CpGs are stratified by whether they are part of a CpG island and whether the associated gene (+- 3kb of the gene body) is predicted to escape from X chromosome inactivation (XCI) (Yang et al., 2010).

Reference:

Yang, F., Babak, T., Shendure, J., and Disteche, C.M. (2010). Global survey of escape from X inactivation by RNA-sequencing in mouse. Genome Res 20, 614-622. 10.1101/gr.103200.109.

Supplemental Figure S4 - Related to Figure 3

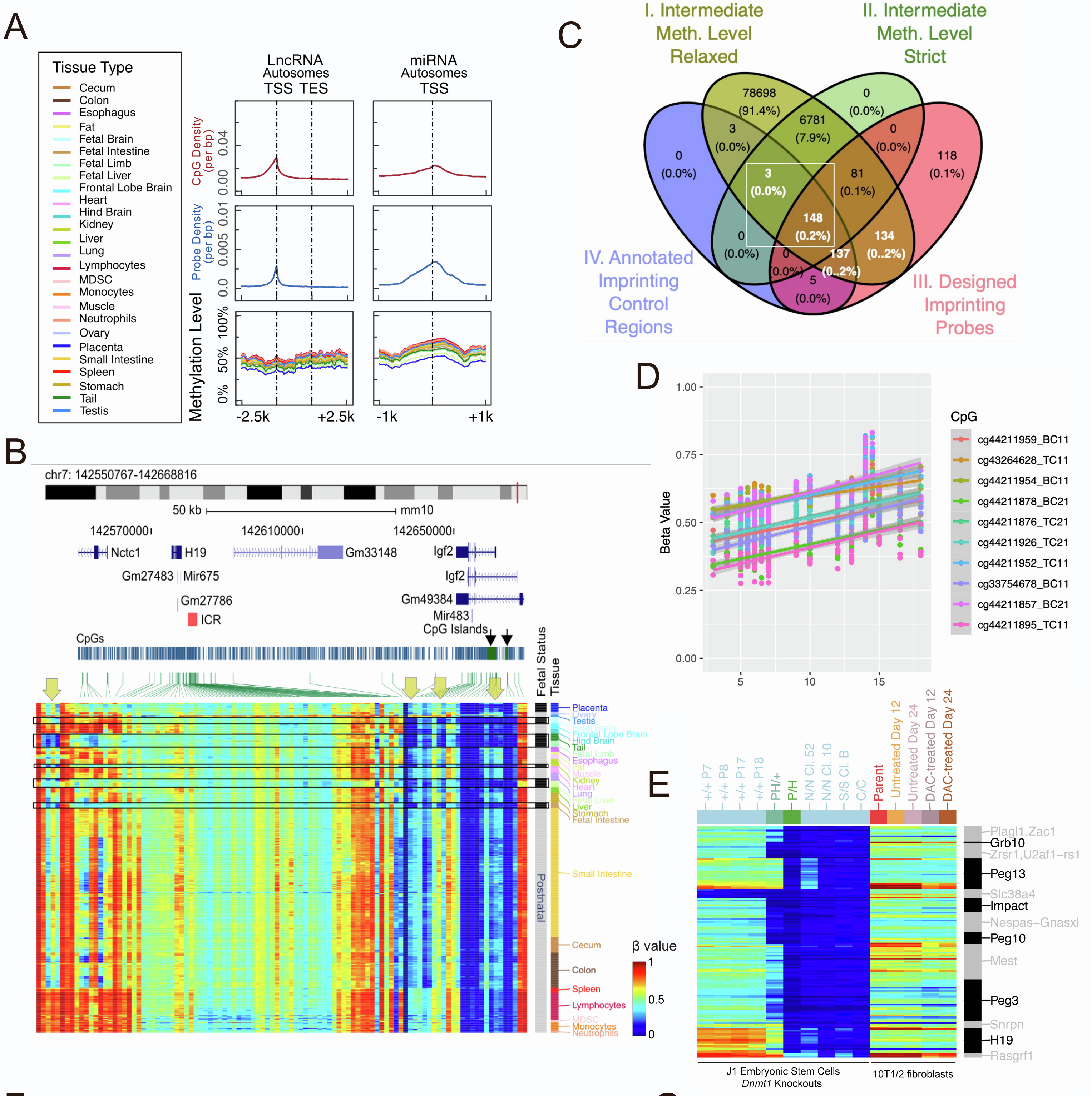

F

	Strain	Tissue	Sex	Age	VMR Count	VMR Frequency (%)	Background Count	Background Frequency (%)	Log2 Fold Change	P-value
All	1	1	1	1	94	1.8	2859	1.1	0.710493383	6.11E-06
Pattern 1	1	0	1	1	16	0.3	383	0.1	1.584962501	5.67E-03
Pattern 2	1	1	0	1	3228	61.5	112800	43.1	0.512898541	2.47E-160
Pattern 3	1	1	0	0	1559	29.7	69452	26.6	0.159036685	2.63E-07
Pattern 4	0	1	0	1	230	4.4	21043	8	-0.862496476	1.51E-26
Pattern 5	0	1	0	0	84	1.6	10100	3.9	-1.285402219	6.27E-22
Pattern 6	1	0	0	1	17	0.3	2421	0.9	-1.584962501	2.64E-07
Pattern 7	0	0	0	1	9	0.2	17545	6.7	-5.06608919	1.06E-142
None	0	0	0	0	8	0.2	20226	7.7	-5.266786541	2.30E-169

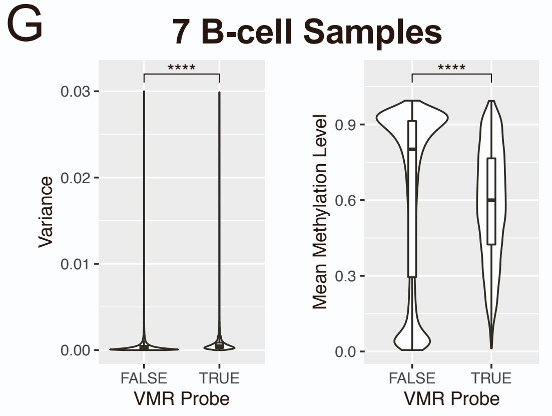

Figure S4. DNA Methylation Analysis of Genomic Features and Regions, Related to Figure 3. (A) Genomic distribution of DNA methylation levels centered on autosomal lncRNAs and miRNAs. The density of CpGs (top row), the density of probes designed for the MM285 array (middle row), and the average methylation level of samples stratified by tissue type (bottom row) are shown accordingly. **(B)** Methylation level of CpGs associated with the Igf2/H19 imprinting region. **(C)** Overlap of four different groups of potential mono-allelic methylation-associated CpGs on the mouse methylation array. Two groups (Group I and II) are based on evidence of consistent intermediate methylation across 138 somatic tissue samples. Group I probes require consistent intermediate methylation in over 50% of the samples (methylation level between 0.3 and 0.7), while Group II requires intermediate methylation in over 90% samples and fully methylated and unmethylated in three testis samples. Sex-chromosome probes are excluded. Group III is imprinting-associated probes designed based on genomic proximity, and Group IV is based on localization of CpG at 13 manually curated imprinting control regions. Probe sets boxed in white are used in the downstream analysis shown in this paper. **(D)** Scatter plot contrasting beta values against age in month in 10 ICR probes most associated with age. **(E)** A heatmap showing DNA methylation level of CpGs (rows) from 13 imprinting control regions in the mouse cell lines, including the J1 embryonic stem cells and the C3H 10T1/2 cells of different *Dnmt1* genotypes with or without DAC treatment. CpGs are ordered by genomic coordinates. The associated imprinting region is labeled on the right. **(F)** Table of the VMR (Variably Methylated Region) probe representation in CpGs for which the methylation level is influenced by strain, tissue, sex, or age (1 indicating an influence, 0 indicating no influence for that covariate). **(G)** Boxplots showing the distribution of the DNA methylation level variance (left panel) and the mean beta value (right panel) of VMR probes compared to non-VMR probes across 7 B-Cell samples (left panel). VMR probes have significantly higher variance and mean beta value compared to non-VMR probes (both P values $< 2.2*10^{-16}$, Wilcoxon rank-sum test).

Supplemental Figure S5 - Related to Figure 4

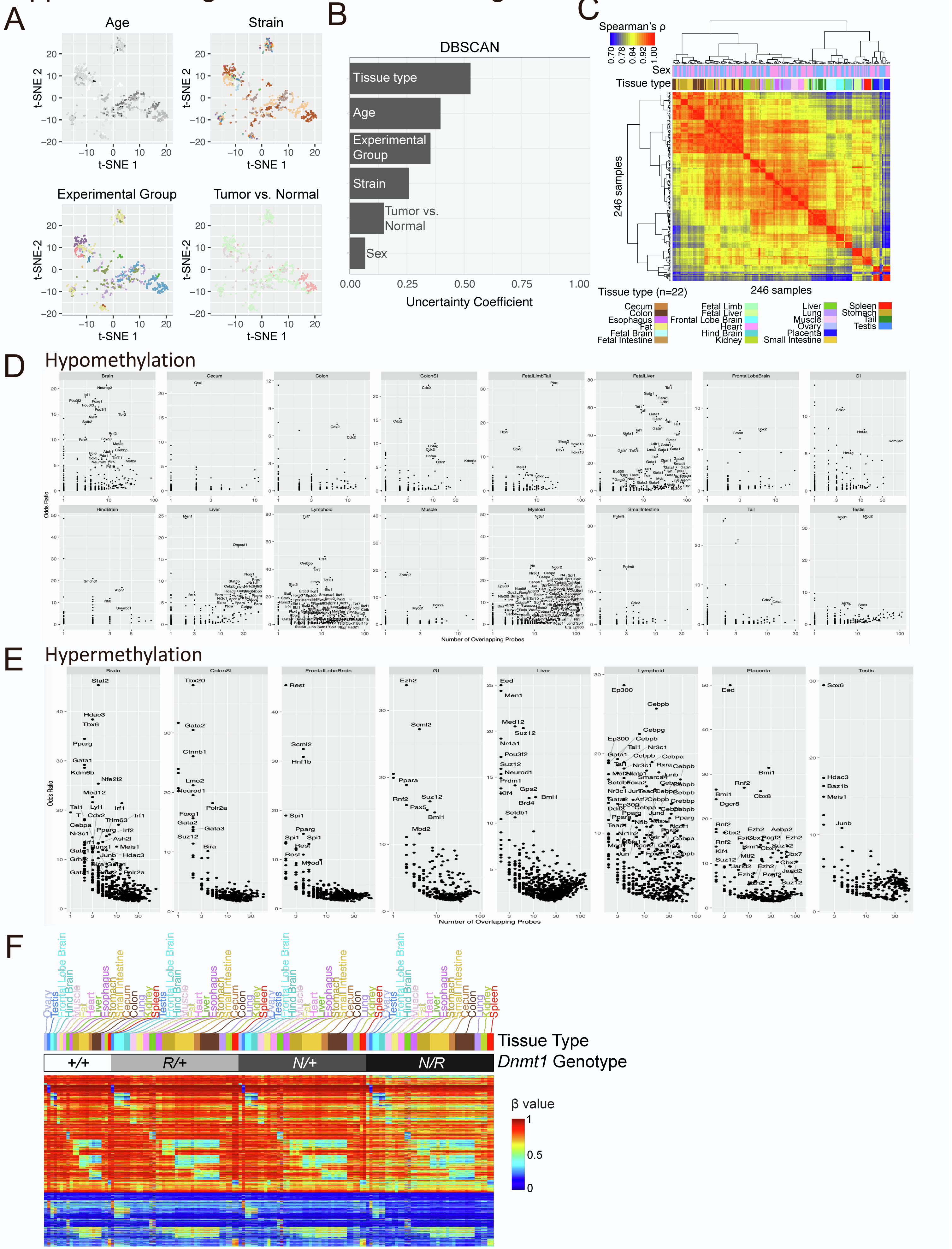

Figure S5. Tissue-Specific DNA Methylation, Related to Figure 4. (A) tSNE cluster map of mouse methylomes colored by tissue, sex, experiment group, strain, cell line state, age, and mean methylation level globally and at Polycomb target genes. **(B)** Uncertainty coefficients of six different sample meta variable predicting DNA methylation-based sample clustering membership. Uncertainty coefficient quantifies the fraction of total information in sample clustering predicted by a random discrete variable. **(C)** Matrix representing hierarchical clustering of pairwise Spearman correlation coefficients of global methylomes of 246 samples representing 22 different tissue types. **(D)** Transcription factors enriched in tissue-specific hypomethylation with odds ratio of enrichment shown on the Y-axis and the number of overlapping probes shown on the X-axis. **(E)** Transcription factors enriched in tissue-specific hypermethylation with odds ratio of enrichment shown on the Y-axis and the number of overlapping probes shown on the X-axis. **(F)** Heatmap of DNA methylation level using tissue-specific probes (rows) in *Dnmt1* hypomorphic mice (columns).

Supplemental Figure S6 - Related to Figure 5

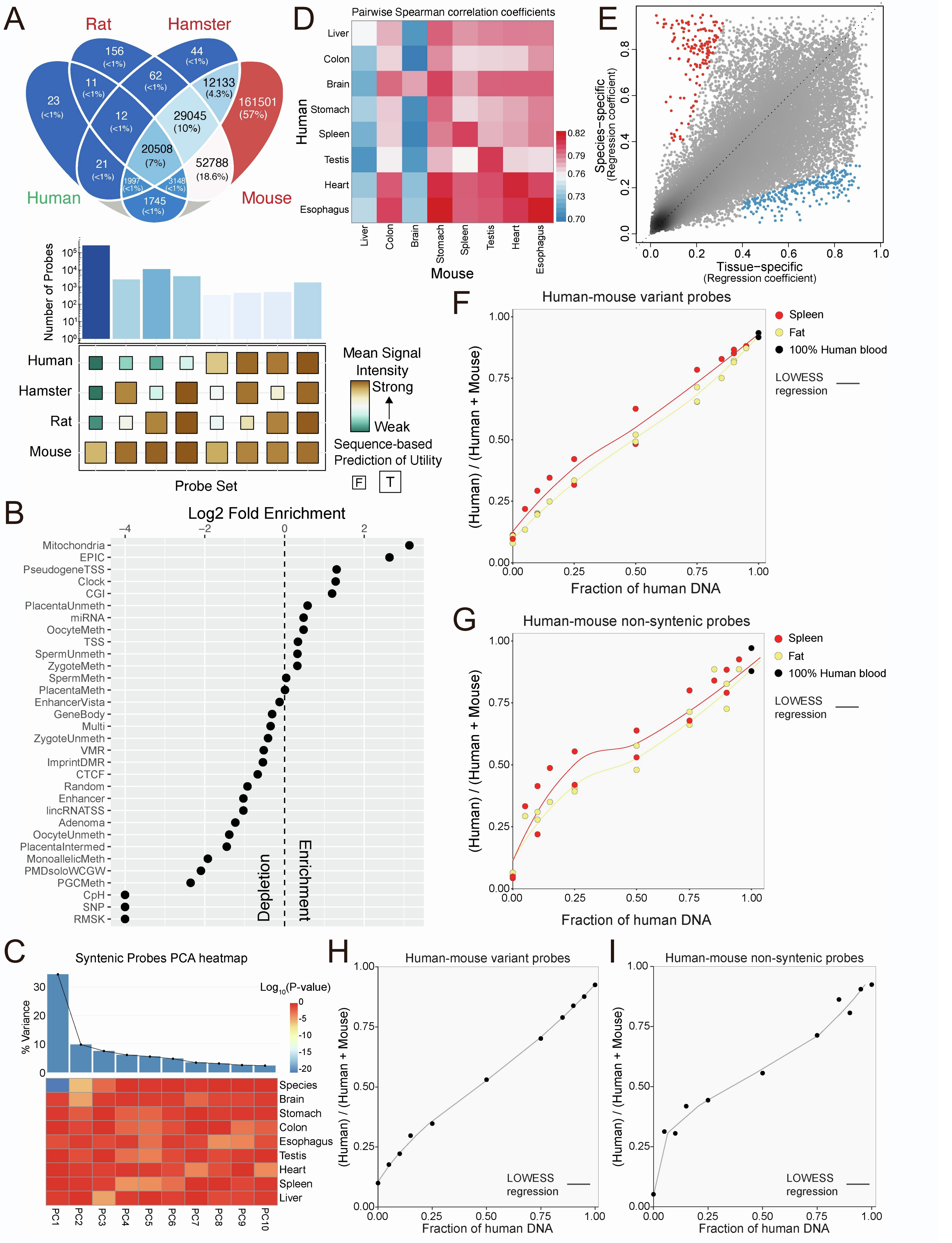

Figure S6. Comparative Epigenomics and Species-Specific Methylation, Related to Figure 5. (A) 4-way Venn diagram showing the predicted probe functionality in human, mouse, rat and hamster genomes. Validation of the mean signal intensity of probes from different sequence-based utility categories for human, hamster, rat, and mouse DNA. Probes are classified by whether they are functional in human, hamster, and rat. Probes are always functional in mouse by design. Strong signal is only observed when the probe category is predicted to work in the corresponding species. **(B)** Enrichment of evolutionarily conserved probes in each design group. Evolutionary conservation is defined by having 60-way PhastCons score greater than 0.8. X-axis plots log2 fold enrichment compared to background probe fraction on the array. Log2 fold enrichment is capped at -4 from the bottom. **(C)** A Heatmap showing the significance (p-value) distinguishing different factors (rows). Wilcoxon rank sum test was used to evaluate the significance of the difference. For tissue, we performed a one-vs-rest pairwise comparison. Percentage of variance explained is shown on top of the heatmap. PC1 is entirely linked to species, while the other PCs are by tissue or a combination of tissue and species. **(D)** Heatmap showing the pairwise Spearman's correlation coefficients of 8 human (rows) and 8 mouse tissues (columns). **(E)** A scatter plot showing the magnitude of tissue-associated variation (X-axis) and species-associated variation (Y-axis) in DNA methylation for each human- mouse syntenic probe (dot). Tissue-specific CpGs (Blue) are defined as probes with delta beta value (regression slope, tissue) > 0.4, delta beta value (tissue) / delta beta value (regression slope, species) > 0.3. Species-specific CpGs (Red) are defined as probes with delta beta value (species) > 0.4, delta beta value (species) / delta beta value (tissue) > 0.3. **(F and G)** LOESS curves fitted between the signal ratios (Y-axis) and the known proportions of human blood DNA mixed in mouse fat (light gold) or spleen (red) DNA samples (X-axis). The signal ratios were calculated using **(F)** the 19 syntenic probes with SNVs at the extension bases between human and mouse and **(G)** the non-syntenic probes in the mouse (n=259,626) and human (n=733,164) arrays. **(H and I)** Standard curves derived using the mean of the two LOESS fitted values from the fat and spleen DNA for the two methods based on **(H)** the syntenic human-mouse variant probes and **(I)** the non-syntenic probes in the mouse and human arrays.

Supplemental Figure S7 - Related to Figures 6 and 7

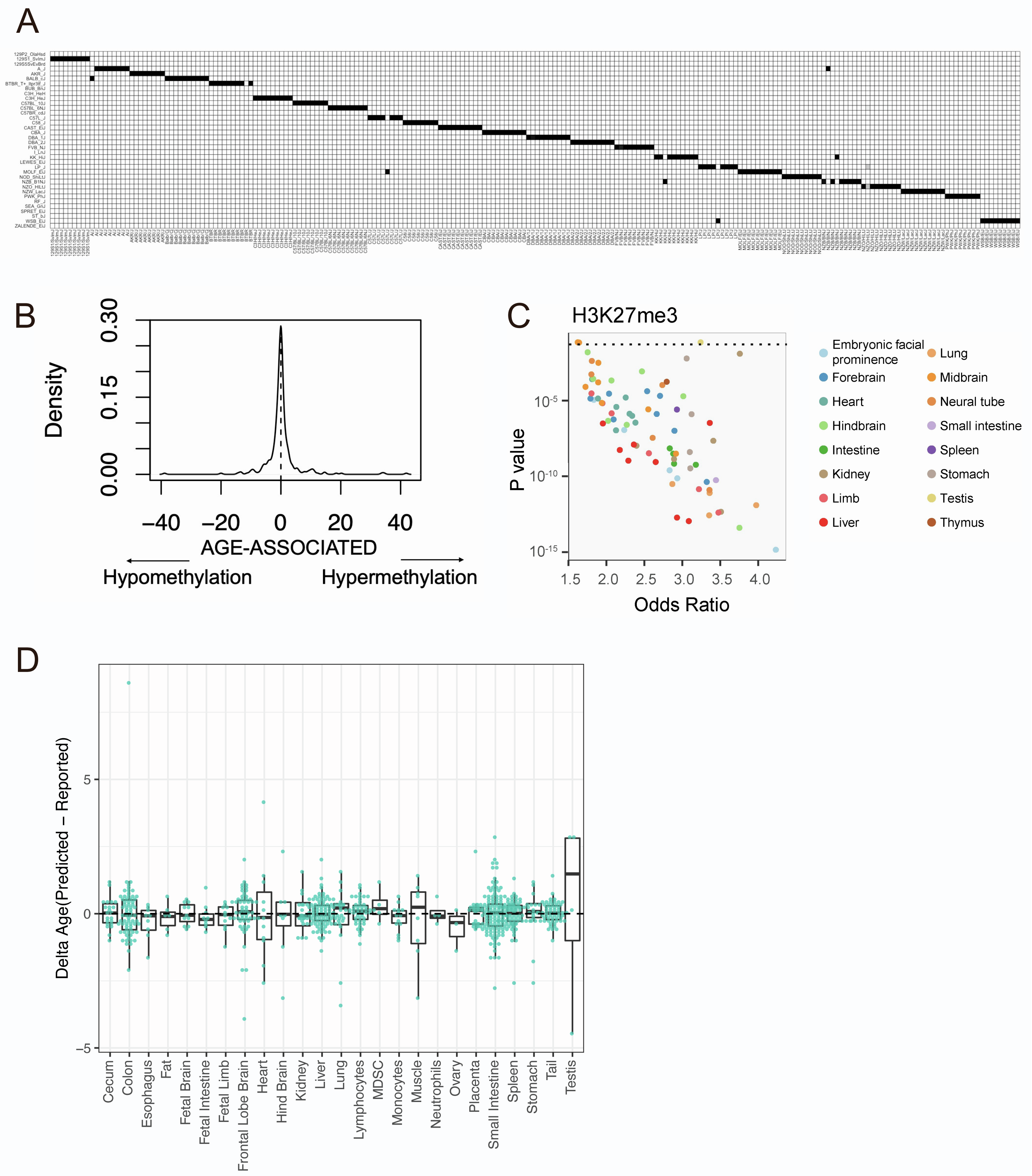

Figure S7. Age-Associated Methylation and Epigenetic Clock, Related to Figures 6 and 7. **(A)** Heatmap showing the likelihood of samples (columns) being predicted to candidate strains (rows) using strain-specific SNPs. **(B)** Distribution of age effect for each probe used in the epigenetic clock, showing roughly equal representation of clock CpGs that gain and lose methylation with age. **(C)** Enrichment of clock CpGs in H3K27me3-marked chromatin. X-axis shows odds ratio and y-axis shows p-value of enrichment. Each dot represents an ENCODE H3K27me3 dataset of a distinct tissue type (color). **(D)** Boxplot showing the distribution of age prediction error stratified by tissue. The figure shows the error is largely unbiased and tissue invariant except for testis for which age tends to be over-estimated.
